# Radio Electric Asymmetric Conveyer (REAC) technology to obviate loss of T cell responsiveness under simulated microgravity

**DOI:** 10.1371/journal.pone.0200128

**Published:** 2018-07-06

**Authors:** Salvatore Rinaldi, Maria Antonia Meloni, Grazia Galleri, Margherita Maioli, Gianfranco Pigliaru, Giulia Cugia, Sara Santaniello, Alessandro Castagna, Vania Fontani

**Affiliations:** 1 Research Department, Rinaldi Fontani Foundation, Florence, Italy; 2 Department of Regenerative and Anti-Aging Medicine, Rinaldi Fontani Institute, Florence, Italy; 3 IRF Shanghai Medical Sciences, Shanghai, China; 4 Department of Clinical and Experimental Medicine, University of Sassari, Sassari, Italy; 5 Department of Biomedical Sciences, University of Sassari, Sassari, Italy; 6 Genetics and Biomedical Research Institute, National Research Council (CNR), Monserrato, Cagliari, Italy; 7 ViroStatic S.r.l., Alghero, Italy; Dartmouth College, Geisel School of Medicine, UNITED STATES

## Abstract

Alterations of the gravitational environment are likely to modify cell behavior. Several studies have proven that T cells are sensitive to gravity alterations and that microgravity conditions may induce immunosuppression and weakened T cell immune response in humans during spaceflights. The aim of this work was to elucidate if a specific treatment of Radio Electric Asymmetric Conveyer (REAC) technology could restore, after mitogenic activation (Con A), a correct expression of cytokine IL2 gene and its receptor IL2R alpha, which are inhibited in T cells under microgravity conditions, as demonstrated in several studies. The results of this study, conducted in microgravity simulated with Random Positioning Machine (RPM), confirm the T cell activation recovery and offer the evidence that REAC technology could contribute to the understanding of T cell growth responsiveness in space, reducing the impact of weightlessness on the immune system experienced by humans in long duration space missions.

## Introduction

The REAC technology (acronym for Radio Electric Asymmetric Conveyor) is a technology platform for neuro- and bio-modulation. Previous studies have proven that REAC technology is able to induce direct cell reprogramming of murine embryonal[1] and human differentiated adult cells toward cardiac, neuronal, and skeletal muscle-like lineages[2, 3]. Moreover, REAC technology has shown to be able to counteract aging processes [4, 5], acting also on telomerase-independent and telomerase-dependent pathways [6] and on endogenous Hyaluronic Acid (HA) and HA-binding proteins. Through its mechanism of action, REAC technology creates an interesting network that acts on the modulation of cell polarity and intracellular environment [7]. On the basis of REAC efficacy as cell polarity optimizer[7], the purpose of this study was the evaluation of REAC technology and in particular of its RGN-S treatment protocol[1–3, 6], as a potential countermeasure to win the impact of spaceflight stress on the alteration of the immune system experienced by humans in the space environment. In fact, one focus of today's research on cells in space is the signal transduction and the underlying mechanism of cell polarity modulation[8].

In the last 30 years, more than 230 experiments conducted in space have shown that dramatic changes occur in several types of cells during their exposure to microgravity, and several studies evidenced microgravity effects onto Immune System and lymphocytes.

T lymphocytes in microgravity were investigated in numerous experiments following Cogoli’s first observation that revealed that the failure of Concanavalin A in stimulating proliferation of lymphocytes was clearly due to the lack of gravity[9]. Concanavalin A activates T Lymphocytes by initiating a complex mechanism, which requires two further signals until the T cells start replicating their DNA. Crucial points of this process are the production of interleukin 2 (IL-2) by T cells and the autocrine interaction of IL-2 with the IL-2 receptor alpha (IL2Rα) expressed at the surface of activated T lymphocytes [10–13]. These experiments concluded that disturbed T cell function in weightlessness is the result of an altered architecture and function of the cytoskeleton, changing the secretion of cytokines and the expression of IL-1/IL-2 receptors[14, 15]. This is why one focus of today's research on cells in space is the signal transduction.

T cells are a good model to study signal transduction pathways, because three extracellular signals (mitogen, IL-1 and IL-2) are required for full activation, and two classical pathways (via proteins G and PKC, PKA) are activated within the cell[16]. In addition, low molecular weight GTP-binding proteins (Ras and Rap) are interacting with the cytoskeleton[15]. The data at 0*g* support the notion that the expression of IL-2 receptor is inhibited, while mitogen binding and the transmission of IL-1 by accessory cells occur normally. Moreover, Hughes–Fulford’s group analyzed induction of early genes expression in Concanavalin A activated human T cells [17, 18] and discovered that the protein kinase A (PKA) signaling pathway is downregulated under microgravity. Transcription factors as NF-κB, AP-1, and CREB are all regulated by PKA and they all suffer dysfunction under altered gravity. These findings indicate that PKA is a key player in gravity-mediated modulation of T cell activation and not just the PKC as believed as far[19].

A systematic approach to understand the causes of the loss of T cell activation was conducted in real microgravity conditions in space and in microgravity conditions simulated by ground facilities, as Fast Rotating Clinostat (FRC)[20] and Random Positioning Machine (RPM)[21, 22]. The results obtained in ground facilities were in agreement with those obtained in space. Therefore, for our work we used the Random Positioning Machine, reproducing the experimental model already used in many studies[23, 24] for the investigation of T cell activation as well as cell differentiation in the immune system[25]. The results obtained revealed that REAC technology effectively reduces the loss of T cell activity in the space and improves the gene expression of IL2 and its IL2-Rα, under simulated microgravity conditions. REAC technology RGN-S treatment protocol could be a potential countermeasure to win the impact of spaceflight stress on the alteration of the immune system experienced by humans in the space environment.

## Materials and methods

### Ethics

The institutional review board of the Local Public Health Authority, the Ethic Committee of Azienda Sanitaria Locale (ASL) N° 1, Sassari, Italy, approved all experimental protocols (Prot. N.2074/CE). Informed consent was obtained from all subjects. The methods were carried out according to the principles expressed in the Declaration of Helsinki.

### Microgravity simulation–The Random Positioning Machine (RPM)

The Random Positioning Machine (RPM) was developed by T. Hoson in Japan and manufactured by Dutch Space (former Fokker Space) in the 1990s[26] to simulate weightlessness, in order to study the effects on plants as well as various other cell types. The RPM provided conditions similar to those that occur during exposure of cells to real microgravity inside an International Space Station (10^−2^/ 10^−4^)[25]. The RPM is an instrument designed to provide an experiment with continuous random orientation changes in 3-dimensional space relatively to Earth’s gravity vector. This three-dimensional movement is achieved by two independently rotating frames. The frames are controlled by a computer, which randomly generates the speed and direction of the frames regardless of the gravity force vector. Biological samples connected to a platform in the middle of the frames experience weightlessness, since they have no time to orient themselves according to the gravity force vector. In our study, the RPM was accommodated in a temperature-controlled room at 37°C, at Biomedical Science Department, University of Sassari, Italy. A box containing the cell cultures sealed in 2 ml Eppendorf tubes, was placed and fixed, as close as possible, at the center of the inner frame of the machine. Eppendorf tubes were used to allow the Asymmetric Conveyer Probe of the REAC device to be immersed in the suspended cells and they were completely filled to avoid the presence of air bubbles, which could lead to shear force damage of the cells on the RPM. A control experiment on ground was also performed: cell cultures, in parallel, were placed at the basement of the RPM, at 1*g* gravity in thermostatic room at 37°C, in static position but continuously turned to prevent them from settling.

### Description of Radio Electric Asymmetric Conveyer (REAC) Technology

Radio Electric Asymmetric Conveyer Technology (REAC) is a technological platform for neuro- and bio-modulation. Its mechanism of action is described in details in Maioli 2016 [7]. REAC is an asymmetric technology since there is only one single physical pole (asymmetrical circuit), while a normal electric circuit has two physical poles: one positive and one negative (symmetrical circuit). This unique pole in REAC Technology is the Asymmetric Conveyer Probe (ACP), which becomes the attractor of the currents induced in the body or in cell culture by the radio electric field emitted by the device. The aim of this scheme is to create an asymmetric circuit, which better interacts with the asymmetric mechanism underlying the cell polarity. Acting on cell polarity and optimizing its functions, REAC technology can modulate the current flows both at cellular and body level, when these are altered, exerting a therapeutic effect. Moreover, in order to induce current flows of intensity comparable with those of cell polarity, REAC Technology uses radio electric emission of low power level, because higher power levels would disturb the adjustment mechanisms of cell polarity. The REAC treatment protocol used in this study was the Regenerative (RGN) treatment type S. The radio electric field was generated by a 2.4 GHz emitter. With the ACP immerged in the cell cultures at a distance of 35 cm from the emitter, we measured a radiated power of approximately 400 μW/m^2^ Electric fieldσ = 0.4 V/m, magnetic field = 1 mA/m, specific absorption rate (SAR) = 0.128 μW/g; given J = 1 A/Vm, and ρ = 1,000 kg/m^3^, the density of radio electric current flowing in the culture medium during a single radiofrequency burst by the REAC is J = 30 μA/cm^2^.The model of REAC device used in this study was B.E.N.E. (Bio-Enhancer Neuro-Enhancer, manufactured by ASMED S.r.l., Florence, Italy).

### Peripheral blood mononuclear cells

For each independent experiment, Peripheral Blood Mononuclear Cells (PBMCs) were separated for every different donor, to overcome the individual variation. Each buffy-coat preparation (45ml) of a blood sample (450 ml) drawn from the antecubital vein of a volunteer healthy donor and collected into blood bag containing citrate phosphate dextrose as anticoagulant (obtained from Centro Trasfusionale, Ospedale Civile–Sassari, Italy), was diluted 1:10 with Hank’s buffer (Gibco) just before the separation. PBMCs were separated as resting cells from the peripheral fresh blood according to the Boyum method[27], based on gradient centrifugation at 300x*g*, by means of the density separation medium Histopaque-1077 (SIGMA) and leading to a cell population consisting of (80%) T lymphocytes, (10%) B cells, (5%) monocytes and (5%) granulocytes, indicated as a whole as Peripheral Blood Mononuclear cells (PBMCs). PBMCs were stored overnight at 37°C inside thermostatic room, in the dark, prior to activation, to avoid causing non-specific activation. This allows the cells to recover from the stress of the isolation. In fact, centrifugation steps lasting 10–30 min at 200x*g* or more may activate certain genes and thus cause artefacts due to hyper gravity stress.

### Cell activation

PBMCs activation can offer a good model for the study of cell differentiation as well as of the cellular aspect of the immune system. For these experiments, PBMCs were resuspended at a density of 6 x 10^6 cells/ml into RPMI-1640 culture medium (GlutaMAXTM, Gibco, Paisley, UK) supplemented with 10% heat-inactivated fetal calf serum (FCS, mycoplasma free, Gibco), 20 mM 4-(2-hydroxyethyl) piperazine-1-ethanesulfonic acid (HEPES), 5 mM sodium bicarbonate, 50 μg /ml gentamycin (all purchased from Gibco, Invitrogen, Carlsbad, USA). Cells were activated by addition of a mitogenic drug, Concanavalin A (Con A, Sigma), at a final concentration of 10 μg /ml and aliquoted into 2ml Eppendorf tubes to a final concentration of 6 x 10^6cells/ml. Con A is a mitogenic drug from lentil seeds of *Canavalia ensiformis* and is known for its ability to stimulate mouse T cell subsets, giving rise to four functionally distinct T cell populations, including precursors to suppressor T cell. Also one subset of human suppressor T cells is sensitive to Con A[28]. Con A was added immediately before the start of the experiment. Upon exposure to mitogen Con A in culture, T lymphocytes can be selectively activated polyclonally to proliferate and to produce a number of lymphokines. T lymphocytes are activated by mitogens of different origin. Most of the results on T cells activation were obtained with the lectin Con A[28].

### Experiment layout of each buffy coat preparation

**Fig 1:**
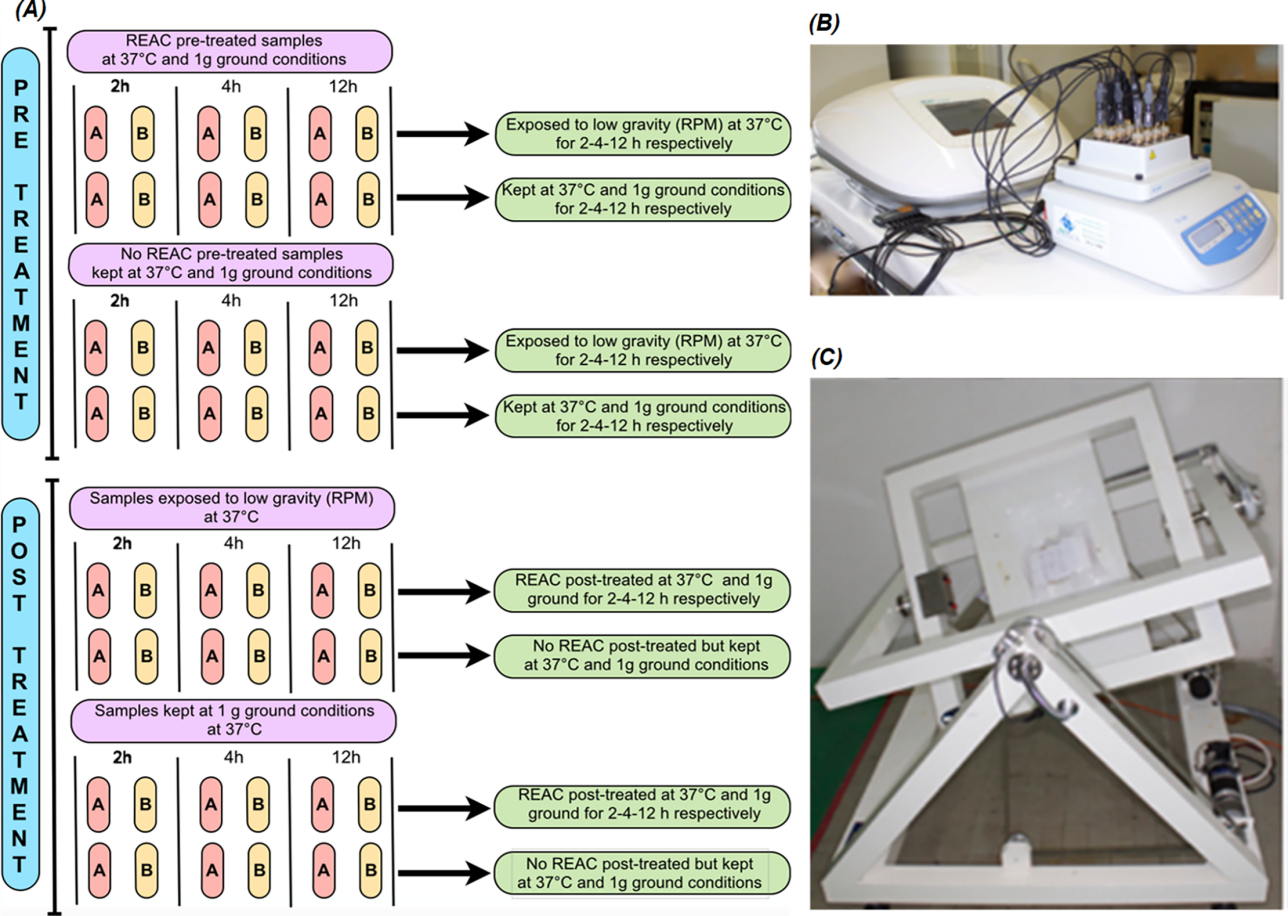
Schematic representation of the experiment profile, REAC treatment of cells and their exposition to simulated microgravity on Random Positioning Machine (RPM) or to 1*g* ground conditions. (A) Flow chart showing the experiment layout of REAC treatment before and after exposition to simulated microgravity (RPM) or 1*g* ground conditions. The Eppendorf tubes were lying on their side. One of the two samples from each experimental set and for each time, named A, was destined for gene expression analysis, placed into ice and subsequently resuspended in 2ml RNAlater; the other sample, named B, was prepared for flow cytometry analysis, centrifuged and after removing cell culture medium, resuspended in freezing medium and subsequently stored at -80°C until processing. (B) Representative image of REAC apparatus during samples treatment at 1*g* gravity. (C) Photographs capturing some steps of cells exposition to simulated microgravity on RPM.

For the REAC treatment experiment, cell culture units were prepared, loading aliquots of 2ml into each culture tube, to provide the samples divided into two experimental sets: REAC pre-treated cells and REAC post-treated cells. REAC pre-treated cells were divided in 6 subsets, each of which consisted of a pair of samples (Fig 1).

The first 3 subsets for each health donor were respectively treated with REAC technology for 2, 4 and 12 hours and then exposed to simulated microgravity (RPM), at 37°C for the same period. The second 3 subsets were respectively treated with REAC technology for 2, 4 and 12 hours and then exposed to 1*g* ground gravity for the same period and in same temperature condition (37°C). Control cells were defined as 6 subsets of cells untreated with REAC: among them, 3 subsets were kept at 1*g* gravity conditions for respectively 2, 4 and 12 hours and then exposed to simulated microgravity for the same period, while the other 3 subsets were kept at 1*g* for respectively 2, 4 and 12 hours and subsequently kept still at 1*g* for the same period. In a similar way, REAC post-treated cells were divided in 6 subsets. The first 3 subsets were exposed to RPM for respectively 2, 4 and 12 hours at 37°C, and then treated with REAC technology for the same period of time. The other 3 subsets were exposed to 1*g* gravity for respectively 2, 4 and 12 hours, and then treated with REAC technology for the same period of time.

As for the control of REAC pre-treatment, even for REAC post-treatment control cells were defined as 6 subsets of cells untreated with REAC: among them, 3 subsets were exposed to simulated microgravity for respectively 2, 4 and 12 hours and then kept at 1*g* gravity for the same period, while the other 3 subsets were exposed to 1*g* ground gravity for respectively 2, 4 and 12 hours and subsequently kept still at 1*g* for the same period. Cell cultures either during REAC pre-treatment or during REAC post-treatment, were placed into a thermostatically-controlled water bath at 37°C, and kept in a static 1*g* gravity condition for each time required (2-4-12 h), as provided by experiment schedule (Fig 1). The culture medium was not changed during the hours of culturing. The samples placed on the RPM bar in 1*g* gravity condition were continuously turned to prevent them from settling. For each experimental subset of cells, one sample was destined for gene expression analysis, placed into ice and subsequently resuspended in 2ml RNAlater. The other sample was centrifuged and after removing cell culture medium, resuspended in freezing medium (10% DMSO in 90% FCS) and subsequently stored at -80°C until staining and acquisition for flow cytometry analysis.

### Gene expression—Quantitative real-time Polymerase Chain Reaction (qPCR)

We selectively performed qRT-PCR to quantitatively detect gene expression of Il-2 and IL2Rα, in order to verify their different expression in REAC-pre-treated cells before exposition to simulated microgravity (RPM) or in REAC-post-treated cells after exposition to RPM, versus their respective no-treated controls either on simulated microgravity (0*g*-RPM) and on 1*g* gravity conditions (1*g*-ground). Cells from each sample of each experimental set and of each time (Fig 1A) were placed into ice and subsequently resuspended in 2ml RNAlater, according to the manufacturer's instruction (Sigma-Aldrich, Milan, Italy). Total RNA was isolated from cells exposed to different experimental conditions, as indicated in experimental profile, using Trizol reagent according to the manufacturer's instruction (Invitrogen, Carlsbad, USA). Total RNA was dissolved in RNAase-free water for RT-PCR and quantified by Nanodrop. The cDNA was synthesized in a 50-μl-reaction volume with 1μg of total RNA and MMLV reverse transcriptase (RT) according to the manufacturer's instruction (Invitrogen, Carlsbad, USA). Quantitative real-time PCR was performed using an iCycler Thermal Cycler (Bio-Rad, Segrate, Italy). 2-μl cDNA were amplified in 50-μl reactions using Platinum Supermix UDG (Invitrogen, Carlsbad, USA), 200 nM of each primer, 10 nM fluorescein (Bio-Rad, Segrate, Italy) and Sybr Green. After an initial denaturation step at 94°C for 10 min, temperature cycling was initiated. Each cycle consisted of 94°C for 15s, 55–59°C for 30s and 60°C for 30s, the fluorescence being read at the end of this step. All the primers used were purchased from Invitrogen, Carlsbad, USA and reported in Table 1.

**Table 1:** Primers used in the study.

GENE	FORWARD	REVERSE
IL-2	CAGGATGCAACTCCTGTCTTG	ATGCTCCAGTTGTAGCTGTGT
IL-2 RA	TTCTCAGCCGCTTCTGACTG	CCTGACATTGCCTCATGGGT
GAPDH	CAGCCTCAAGATCATCAGCA	TGTGGTCATGAGTCCTTCCA

To evaluate the quality of product of real-time PCR assays, melting curve analysis was performed after each assay (data not shown). Relative expression was determined using the “delta-CT method” with GAPDH as reference gene[29].

### Flow cytometry

Flow cytometry analysis of Peripheral Blood Mononuclear Cells (PBMCs) collected at different time intervals were performed for each sample. In particular, one of the two samples from each experimental set and for each time (Fig 1B) was destined to flow cytometry. Cells from each sample were centrifuged and after removing cell culture medium, were resuspended in freezing medium (10% DMSO in 90% FCS) and subsequently stored at -80°C and they were left until staining.

### Cell viability assay

Before the experiment, Trypan blue exclusion test was made, as isolation laboratory “routine” test, in order to assess cell viability. This dye exclusion test is used to determine the number of viable cells present in a cell suspension. Cell suspension is simply mixed with dye and then visually examined to determine whether cells take up or exclude dye, so that a viable cell will have a clear cytoplasm whereas a nonviable cell will have a blue cytoplasm.

Moreover, after cells thawing, we stained with live/dead cell marker 7-aminoactinomicyn-D (7AAD, from Becton Dickinson (BD), San Jose, CA, USA) in the presence of Annexin V, which evaluates PBMCs apoptosis. Then cells were analyzed using flow cytometer FACS CANTOII (BD Biosciences) and, in the "gating" strategy, the non-colored cells with either 7AAD (dead) or with Annexin V (apoptotic) were considered alive, by exclusion. A total of 30.000 events for each sample were acquired and data were analyzed using the Diva 6.2 software (Becton-Dickinson).

### Protein expression analysis

Live cells were quickly thawed in water at 37° C before staining and washed once in phosphate buffer saline (PBS) at 37° C inside thermostatic room. For each sample, 2×10^6 cells were added with the mix of antibodies in a 100μl final volume of FACS flow buffer, for the staining, and incubated for 30 min in the dark. After staining, samples were washed adding 2mL of FACS flow solution, centrifuging for 10 min at 1200 rpm at 37°C and discarded the supernatant by inversion. Then they were resuspended in 300 μl of FACS Flow buffer and the samples were ready for acquisition in flow cytometry. No single T cells were tested but PBMC, a mixture of immune cells in order to maintain intact the conditions of physiological interaction between the immune cells. CD3, CD4, CD8 and CD14 were not the target of our study, but they have been used only in the population isolation T-cell gating strategy in FCS, but they were not analyzed. Stained cells, after washing, were analyzed on a FACS CANTOII (Becton-Dickinson) flow cytometer; a total of 10.000 events for each sample were acquired and the data were analyzed using the Diva 6.2 software (Becton-Dickinson). Unstained cells underwent staining controls as well.

The chemicals used in this study came from the following sources: mAb CD25-phycoerythrin-cyanine (PE-Cy7) was used to detect IL2Rα on the membrane protein.

To determine the phenotype of cellular subpopulations of PBMCs, CD3-fluorescein isothiocyanate (FITC), CD14-phycoerythrin (PE), CD4-alloficocyanine (APC), D8-alloficocyanin-7 (APC-Cy7) were used. 7-Aminoactinomycyn-D (7AAD), BD (Becton Dickinson Biosciences, San Jose, CA, USA) and Annexin V-FITC were used by Life Technologies (Grand Island, NY, USA) for apoptosis analysis. In addition, Fetal Calf Serum (FCS) and Dimethyl sulfoxide (DMSO) were from Gibco Life Technologies (Grand Island, NY, USA) as long as they were purchased. All buffers and other solutions were prepared from analytical grade chemicals available locally.

Flow cytometry analysis was performed by means of a FACSCANTOII (Becton Dickinson, San Jose, CA, USA), flow cytometer equipped with a three-laser excitation system.

### Statistical analysis

Comparisons of outcome data during time were performed. The statistical analysis of the data was performed by using the Graph Pad Prism 5 software. For this study, descriptive analyses included the computation of means, standard deviation (SD) and paired Student’ t test to evaluate the distribution and homogeneity of variance of each group at different times of observation and subsequent Wilcoxon test to evaluate the variant for two independent groups. Tests and all results p<0.05 have been considered statistically significant.

## Results

In this study three independent experiments for each of three donors were performed.

The data shown in this paper are consistent with three analogous experiments.

In the scheme of Fig 1 is shown the layout of a single experiment.

### Effects of REAC-RGN-S treatment on cells apoptosis

**Fig 2:**
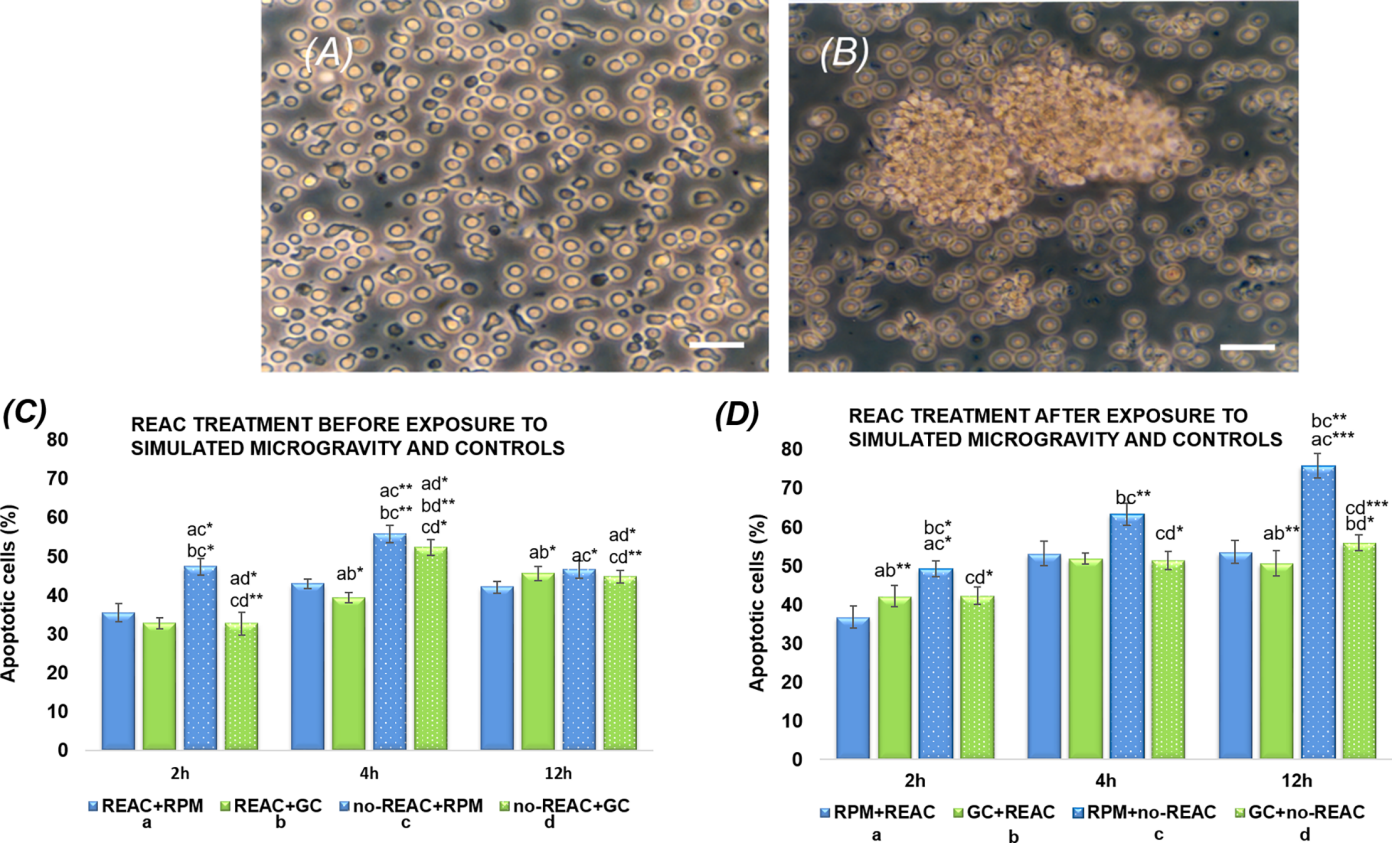
Morphology of PBMCs not activated and activated and subjected to simulated microgravity and analysis of REAC RGN-S treatment effect on cell death programming. **(A)** PBMCs not treated with mitogen, simulated microgravity exposure for 12h; **(B)** The cells show typical activation clusters of mitogenic treatment, exposed to simulated microgravity (scale bar represents 100 μm). **(C-D)** Apoptosis analysis in pre- and post-REAC treated cells and in noREAC treated cells, in simulated RPM microgravity conditions or in Ground Control (GC), at different experimental time (2-4-12 h). **(C)** The graph shows the total apoptosis of cells post-treated with REAC or untreated, and subsequently exposed to RPM (blue bars) or left to 1*g* (GC) (green bars) at different experimental time (2-4-12 h). With lowercase letters the significance ratios are indicated respectively between REAC+RPM (a), REAC+GC (b), noREAC+RPM (c) and noREAC+GC (d). In particular, at experimental times of 4 h and 12 h apoptosis is significantly lower in REAC+RPM compared both to noREAC+RPM (4h p <0.001; 12h p <0.01) and noREAC+GC (4h p <0.001).

REAC-RGN-S treatment had no toxic effect on Peripheral Blood Mononuclear Cells (PBMCs). In general, in the percentage values of graphs C and D (Fig 2), we can observe that REAC treatment influences positively the cells exposed to simulated microgravity, reducing apoptosis compared to the untreated samples in simulated microgravity.

It is important to note that REAC pre-treatment prevents the apoptotic effect of simulated microgravity even at 2h, as shown in the comparison between REAC+RPM and noREAC+RPM (p <0.01). This difference is not observed in the comparison between REAC+GC and noREAC+GC at 2h. (D) The graph shows the total apoptosis of cells post-treated with REAC or untreated, and previously exposed to RPM (blue bars) or left to 1*g* (GC) (green bars) at different experimental time (2–4–12 h). With lowercase letters the significance ratios are indicated respectively between RPM+REAC (a), GC+REAC (b), RPM +noREAC (c) and GC+noREAC (d). In post-treatment, the effect of REAC is more evident, especially in the samples exposed to microgravity in comparison to Ground Control. The difference between RPM+REAC and RPM+noREAC is statistically significant (2h p <0.001; 12h p<0.0001). In RPM+REAC sample, apoptotic cells decrease about 20% at 12 h, compared to RPM+noREAC. In samples left at 1*g*, no difference is observed between REAC or noREAC post-treated cells. This show that REAC is more effective where there is a insult damage. In the apoptosis plots, the data describe the total cells in apoptosis, with mean ± SD (with n = 3) *p <0.01, **p <0.001, ***p <0.0001, compared all against all. The data were analyzed by DIVA 6.1 software (BD).

### Effect of REAC RGN-S on IL2Rα and IL2 gene expression of cells pre-treated before exposition to simulated microgravity model (RPM) or post-treated after exposition to RPM

**Fig 3:**
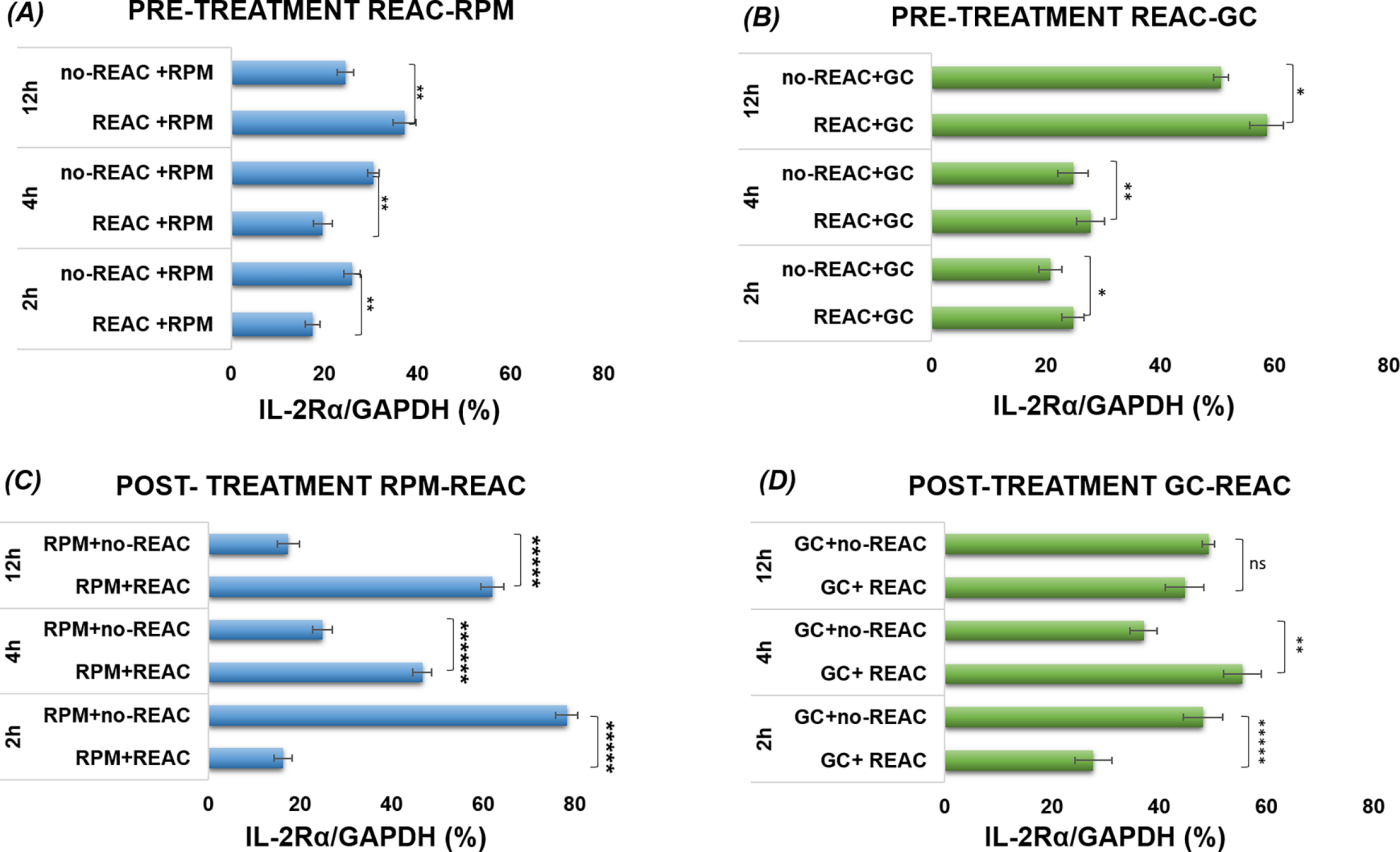
Quantitative real-time qPCR: REAC/microgravity. (A) IL2Rα gene expression at different time (2-4-12h) of cells pre-treated with REAC before exposition to RPM simulated microgravity (REAC+RPM) for 2-4-12h in comparison to noREAC treated cells before exposition to RPM simulated microgravity (noREAC+RPM); (B) REAC pre-treated cells subsequently kept for 2-4-12h at 1*g* gravity conditions (REAC+GC), compared to 1*g* gravity conditions not REAC pre-treated (noREAC+GC). (C) IL2Rα gene expression at different time (2-4-12h) of REAC post-treatment after exposition to RPM simulated microgravity (RPM+REAC) for 2-4-12h, compared to REAC untreated cells after exposition to RPM simulated microgravity (RPM+noREAC). (D) REAC post-treated cells after exposure to 1*g* gravity conditions (GC+REAC) compared to 1*g* gravity conditions without REAC post-treated (GC+noREAC). To evaluate the quality of product of real-time PCR assays, melting curve analysis was performed after each assay (data not shown). Relative expression was determined using the “delta-CT method” with GAPDH. Data are expressed as mean ± S.D. of independent triplicate samples for each treatment.

**Fig 4:**
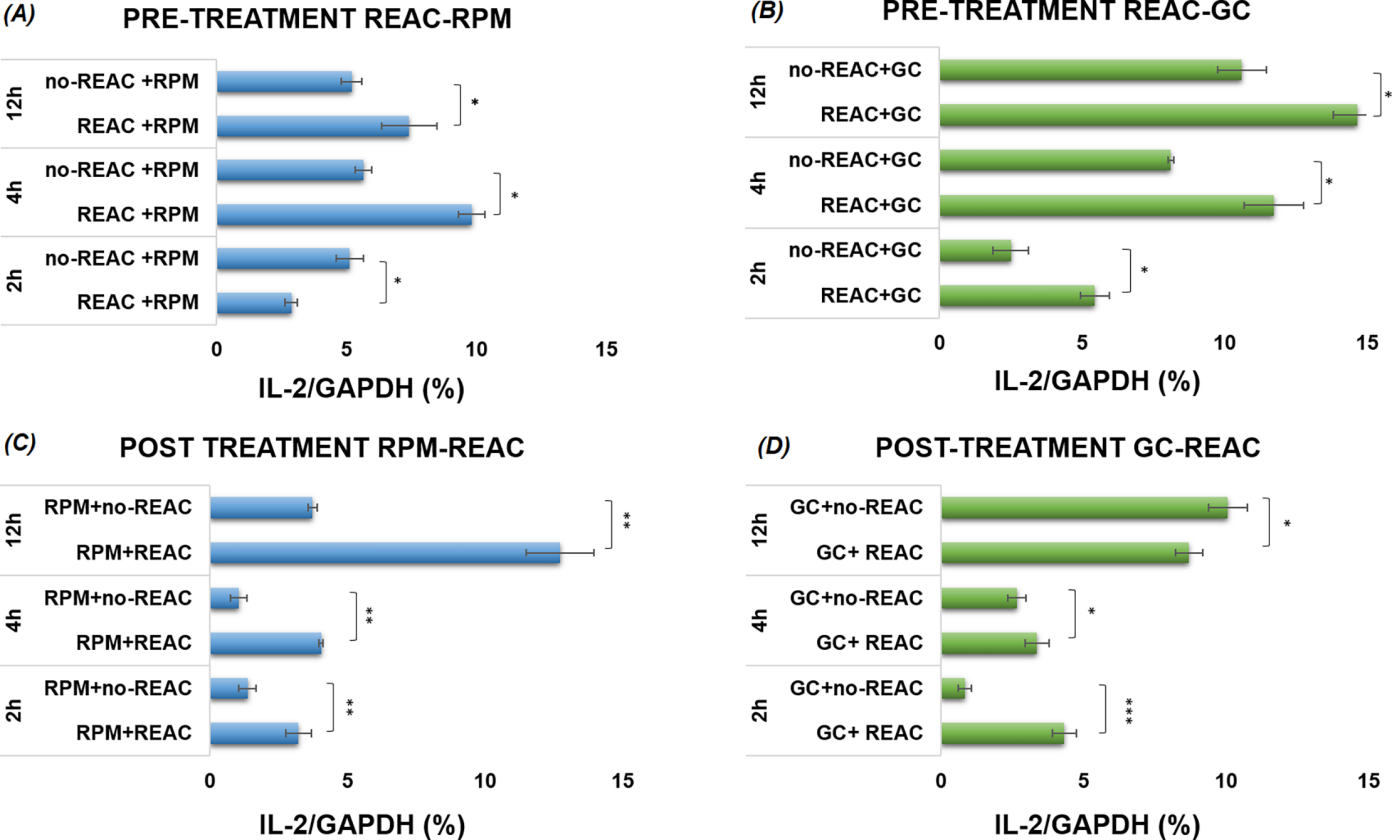
Quantitative real-time qPCR: REAC/microgravity. (A) IL2 gene expression at different time (2-4-12h) of cells pre-treated with REAC before exposition to RPM simulated microgravity (REAC+RPM) for 2-4-12h in comparison to noREAC treated cells before exposition to RPM simulated microgravity (noREAC+RPM); (B) REAC pre-treated cells subsequently kept for 2-4-12h at 1*g* gravity conditions (REAC+GC), compared to 1*g* gravity conditions not REAC pre-treated (noREAC+GC). (C) IL2 gene expression at different time (2-4-12h) of REAC post-treatment after exposition to RPM simulated microgravity (RPM+REAC) for 2-4-12h as compared to REAC untreated cells after exposition to RPM simulated microgravity (RPM+noREAC). (D) REAC post-treated cells after exposure to 1*g* gravity conditions (GC+REAC) compared to 1*g* gravity conditions without REAC post-treated (GC+noREAC). To evaluate the quality of product of real-time PCR assays, melting curve analysis was performed after each assay (data not shown). Relative expression was determined using the “delta-CT method” with GAPDH. Data are expressed as mean ± S.D. of independent triplicate samples for each treatment.

REAC-RGN-S affects IL2Rα and IL 2 gene expression. Figs 3 and 4 show the effect of REAC-RGN-S treatment on the mRNA levels of both IL2Rα (Fig 3) and IL2 (Fig 4).

### Effect of REAC treatment on IL2Rα gene expression

As regards REAC pre-treatment (Fig 3A), we observed that the onset of genetic expression of the IL-2Rα chain in Con A-activated cells begins at a lower level approximately 2 hours after exposure to the mitogen; up to 4 hours, the amount of mRNA remained unchanged and there was no significant difference in the different experimental conditions. Starting from 12 hours, IL-2Rα mRNA levels of REAC treated cells followed by RPM simulated microgravity (REAC+RPM) were increased compared to untreated cells (noREAC+RPM) (p< 0.001). It was also observed that treated samples at 1*g* (REAC+GC) (Fig 3B) showed IL-2Rα gene expression statistically significant higher than noREAC+GC sample at all experimental time (2h p<0,01; 4h p<0.001 and 12h p<0.01). We conclude that REAC pre-treatment induces an increase in IL-2Rα gene expression in cells subsequently cultured under simulated microgravity at 12 hours. In fact, REAC pre-treatment seems to improve the adaptation to simulated microgravity in samples exposed to RPM (REAC+RPM) compared to untreated samples also exposed to simulated microgravity (noREAC+RPM) (p <0.001), in early experimental time (2, 4 h). As regards the REAC post-treated subsets of cells (Fig 3C), we observed that cells exposed to simulated microgravity and subsequently REAC treated (RPM+REAC) showed an increase of IL-2Rα mRNA levels starting from 4 hours up to 12 hours in comparison with untreated samples that show a higher level of mRNA at the earliest experimental 2 hours activation time but the lowest at 12 hours (RPM+noREAC) (p< 0,0000001). (Fig 3D) No difference was observed between samples kept at 1g gravity and then REAC treated (GC+REAC) or untreated (GC+noREAC) at 12h, whereas we observed a significative difference at 4h (p<0.001). The same trend was also observed with respect to IL 2 post- treated samples (Fig 4C and 4D), showing that gene expression is not influenced by REAC treatment in samples before kept at 1g gravity conditions during their activation. It can be deduced that REAC treatment is more effective in cells subjected to a stress during their activation, as altered gravitational environment, (RPM+REAC) compared to untreated cells (RPM+noREAC).

### Effect of REAC treatment on IL2 gene expression

In another set of experiments, we evaluated the effect of REAC-RGN-S treatment after culturing cells in simulated microgravity conditions for 2, 4 and 12 hours (REAC-post treatment in Fig 4C and 4D. Fig 4A and 4B shows the effect of REAC treatment on IL-2 gene expression in cells REAC treated before exposition to simulated microgravity (RPM) or normal gravity condition (GC). REAC pre-treated cells subsequently exposed to simulated microgravity (REAC+RPM) exhibited a higher level of IL-2 mRNA at 4 hours of culture in simulated microgravity, being still higher also at 4,12 hours of simulated microgravity, as compared to the correspondent REAC untreated cells (noREAC+RPM) (p<0.01). Also in this case, as expected according to our previous reports, IL-2 mRNA levels are decreased in RPM untreated cells (noREAC+RPM) in later experimental time (4,12h). As regards REAC post-treatment effect, Fig 4C and 4D shows IL-2 gene expression analysis on cells cultured in different simulated microgravity conditions and then treated with REAC technology. The genetic expression of the IL-2 begins at a lower level approximately 2 hours after exposure to the mitogen; up to 4 hours, the amount of mRNA remained unchanged also there was significant difference in the different experimental conditions compared to untreated sample (RPM+noREAC). Starting from 4 hours and much more at 12 hours, IL-2 mRNA levels of REAC treated cells after RPM simulated microgravity exposure (RPM+REAC) was highly increased compared to untreated cells (RPM+noREAC) (p< 0.001). On the other hand, we observed that the difference between REAC post-treated and untreated cells cultured in normal gravity condition (GC+REAC) (GC+noREAC) decrease during the time course analyzed from 2 at 12h where the conditions REAC untreated (GC+noREAC) were a few increases than GC+REAC (P<0.05). Considering these results, we can conclude that REAC treatment can significantly modify both IL-2 and IL-2Rα gene expression and rescue the inhibitory effect induced by microgravity, in manner time dependent.

### Effects of REAC treatment on IL2Rα protein expression in cells REAC pre-treated before exposition to RPM simulated microgravity

**Fig 5:**
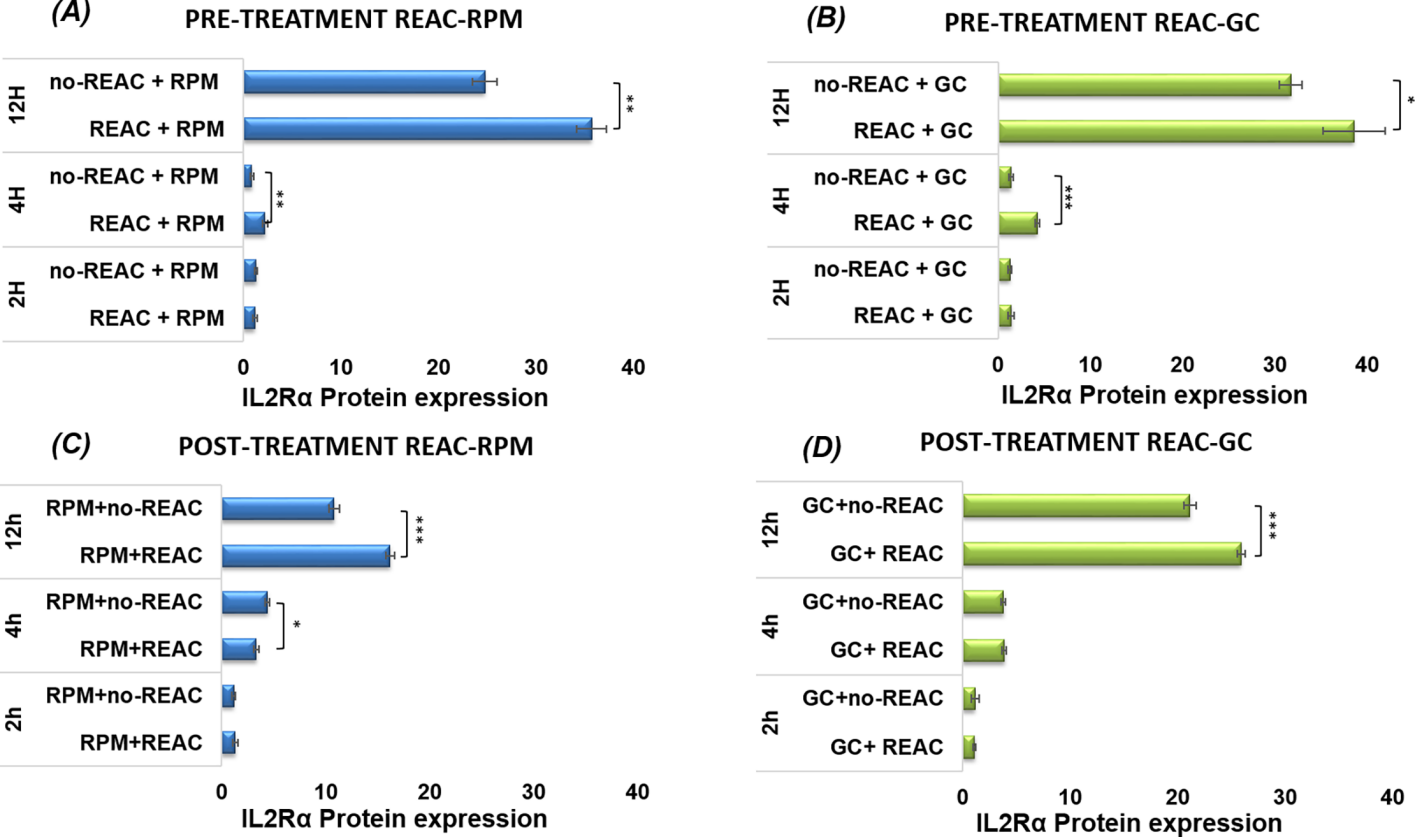
IL2Rα protein expression by Flow cytometric analysis. (A) IL2Rα gene expression at different time (2-4-12h) of cells pre-treated with REAC before exposition to RPM simulated microgravity (REAC+RPM) for 2-4-12h in comparison to no-REAC-treated cells before exposition to RPM simulated microgravity (noREAC+RPM); (B) REAC pre-treated cells subsequently kept for 2-4-12h at 1*g* gravity conditions (REAC+GC), compared to 1*g* gravity conditions not REAC pre-treated (noREAC+GC). (C) IL2Rα cell expression percentage at different time (2-4-12h) of REAC post-treatment after exposition to RPM simulated microgravity (RPM+REAC) for 2-4-12h as compared to REAC untreated cells after exposition to RPM simulated microgravity (RPM+noREAC), (D) REAC post-treated cells after exposure to 1*g* gravity conditions (GC+REAC) compared to 1*g* gravity conditions without REAC post-treated (GC+noREAC). Data are expressed as mean ± S.D. (n = 3, *p<0.01; **p<0.001; ***p<0.0001) of independent triplicate samples for each treatment.

REAC pre-treated cells (REAC+RPM) at different time (2-4-12h) before exposition to RPM simulated microgravity for 2-4-12h demonstrated an evident increase of the cell percentage expressing IL2Rα protein compared to respective untreated cells (noREAC+RPM) (Fig 5A).

The benefit of the REAC pre-treatment can be observed already between 2h-4h and increases after 4h (2.24±0.25 vs. 0.9±0.2, p<0.001), in particular up to 12h (35. 57±1.5 vs. 24.88±1.2, p< 0.001), which is the "end point" of the experiment when Peripheral Blood Mononuclear Cells (PBMCs) have a sudden increase in the expression of IL-2Rα. REAC pre-treatment effect increased after 4h, namely the moment of increased expression of cell membrane markers induced in response to the mitogenic stimulus of activation. It can be stated that REAC pre-treatment for 2-4-12h has a better effect on cellular system subsequently exposed to an environmental stress such as the simulated microgravity (REAC+RPM), compared to untreated one (noREAC+RPM). Yet, REAC can also improve the performance of cells subsequently kept for 2-4-12h at 1*g* gravity conditions at the time of expression of the IL2Rα (REAC+GC), in comparison with respective ground control REAC untreated cells (noREAC+GC). In particular, we can observe a significant increase of IL2Rα expression percentage already after 4h (4.32±0.26 vs. 1.3±0.2, p<0.001). Moreover, in the REAC untreated samples (no REAC+GC and noREAC+RPM), that were our controls in the processing, cell expression of IL2Rα showed no differences between 2 and 4h, it starts to increase after 4 hours (1.3±0.2 vs. 0.9±0.2, p<0.01), grows up to 12h (31.87±1.24 vs. 24.88±1.23, p< 0.001) and it is greater at 1*g* conditions (noREAC+GC), if compared to RPM simulated microgravity (noREAC+RPM). These results are in agreement with our previous data[17, 18, 24, 30]. Our results demonstrated that REAC pre-treatment has a better effect on cellular systems subjected to environmental stress, such as simulated microgravity, though it can also improve the performance of not stressed systems.

### Effects of REAC treatment on IL2Rα protein expression on cells exposed to RPM simulated microgravity model and post-treated with REAC

The REAC post-treatment occurs in the cellular system already activated for the clonal proliferation on RPM simulated microgravity or at 1*g* gravity conditions after different times, 2-4-12h (Fig 5C). As a whole, all samples showed no differences on IL2Rα protein expression between 2h and 4h, while IL-2Rα receptor expression started to increase from 4 hours up to 12 hours. In particular, it has been observed that cells exposed to RPM simulated microgravity before REAC treatment (RPM+REAC) showed a greater expression of IL2Rα at 12h in comparison with respective untreated cells after exposition to RPM (RPM+noREAC) (16,33±0,41 vs. 10.6±0.53, P < 0.0001). It can be stated that if the cells were subjected to a stress during their activation, as altered gravitational environment (RPM+REAC), the effect of REAC treatment is positive in respect of the activation parameters, such as IL2Rα, which is one of the first molecules expressed during the Peripheral Blood Mononuclear Cells activation, when compared to REAC untreated cells (RPM+noREAC). (Fig 5D) On the other hand, if the cellular system does not undergo any stress following the activation stimulus (GC+REAC), REAC treatment shows to be ineffective if compared to REAC untreated ground control (GC+noREAC) (21.28±0.37 vs. 25.63±0.5, p< 0.001).

## Discussion

Immunosuppression and weakened T cell immune response during spaceflight are a major barrier to safe, long-term human space habitation and travel[25]. All this can be counteracted by a good maintenance and modulation of T cell polarity[31]. It is well known that in vivo activation of naive or resting T cells initiated by the binding of a foreign antigen, can be mimicked in vitro by the use of several agents or by concanavalin A (Con A) alone, as activator of T cells, in Peripheral Blood Mononuclear Cells[28]. Upon exposure to mitogen Con A in culture, PBMCs can be selectively activated polyclonally to proliferate and to produce a number of lymphokines. In particular, when exposed to Con A for at least 2 h, they will transcribe and secrete interleukin-2 (IL-2). The regulation of the transcription of the IL-2 gene is based on complex and still obscure mechanisms involving transcription factors such as NFAT, AP-1, NF-kB and others, and it is object of extensive studies, extensively reviewed[32, 33]. The protein expression of immediate-early genes, within 30 min of activation, in turn activates IL-2 expression[34, 35]. The expression of IL-2 induces synthesis of IL-2Rα; both are essential for efficient immune response, T cell proliferation and progression from the G0 to G1 phase[36, 37]. The low gravity environment of spaceflight has proven to lead to impaired T cell activation and profoundly down regulated transcription[30] of immediate early genes. These results demonstrated and confirmed that reduced gravity can perturb molecular signals leading to impaired immune function. DNA array analysis of T cells subjected to RPM microgravity revealed an alteration of several signal modules, in particular NF-kB and MAPK-signaling [17, 33]. A number of potential, gravity-sensitive mechanisms are implicated in the impairment of T cell activation and proliferation in spaceflight, but they are at least in part a result of decreased IL-2 and IL-2Rα induction. We examined gene expression and synthesis of IL-2 as well as IL-2Rα subunits at early time points following activation and characterized the global gene expression of activated T cells in micro-gravity after 4h of activation[17]. This time-point was determined to be the peak for IL-2 expression post activation. In fact, significant up regulation of IL-2 and IL-2Rα was observed during T cell activation by 4h, while there was significant inhibition in micro-gravity[17, 18]. We also studied the time course of gene expression after T cell activation and the pattern of how micro-gravity inhibits the first wave of immediate early genes with downstream effects on a wider range of secondary response genes. Among genes found to be down-regulated significantly in simulated micro-gravity, compared with 1*g*, which became detectable at 4h of activation, are included IL-2 IL-2Rα[30]. The goal of the present experiments was to verify if REAC-RGN-S treatment could be an effective countermeasure against the loss of T cell activity in space. Based on the results of this paper, the most important conclusion we can draw is that if T cells suffered a stress, such as weightlessness, during their activation, the effect of REAC-RGN-S treatment is positive in respect of the activation parameters. We can hypothesize that REAC acts as a T cell polarity optimizer[7], remodulating the cell signal transduction pathways altered by reduced gravity, probably acting on gravity-sensitive cellular targets, where the lipid-raft-associated membrane-proximal signalosome complex is located[33]. Acting on ion fluxes at molecular level, REAC is likely to affect the release of intracellular stored calcium in the endoplasmic reticulum, involved in the activation of the protein kinase C (PKC). REAC effect on stored calcium is likely to be involved also in the regulation of lymphocyte locomotory activity, which under microgravity culture conditions is inhibited, as calcium signal was shown to be a directionality marker for the orientation of neutrophils locomotion[38, 39]. The extensive research on immune cells in culture under different gravity conditions and treated with REAC technology could contribute significantly to the understanding of T cell growth responsiveness in space and may aid in the individuation of a potential countermeasure to win the impact of weightlessness on the alteration of the immune system experienced by humans during long duration missions.

## Supporting information

S1 Data Data collected in the study to assess the effect of REAC treatment on IL2Rα and IL2 gene expression in cells exposed to RPM low gravity model. (XLSX)
